# Transperineal Ultrasound Comparison of Longitudinal Sling Position and Dynamic Sling Configuration After Altis and MiniStop Single-Incision Mini-Slings for Stress Urinary Incontinence

**DOI:** 10.3390/biomedicines14102388

**Published:** 2026-10-19

**Authors:** Liang-Ying Ke, Cheng-Yu Long

**Affiliations:** 1 Department of Obstetrics and Gynecology, Kaohsiung Medical University Hospital, Kaohsiung Medical University, 100, Shih-Chuan 1st Road, Kaohsiung 80708, Taiwan; 2 Department of Obstetrics and Gynecology, College of Medicine, Kaohsiung Medical University, Kaohsiung 80708, Taiwan; 3 Department of Obstetrics and Gynecology, Kaohsiung Municipal Siaogang Hospital, Kaohsiung Medical University, Kaohsiung 80708, Taiwan

**Keywords:** transperineal ultrasound, stress urinary incontinence, single-incision mini-sling, midurethral sling, urodynamics, patient-reported outcomes

## Abstract

**Background/Objectives:** Direct imaging comparisons of single-incision mini-slings are limited. This study compared six-month three-dimensional transperineal ultrasound (TPUS) findings after Altis and MiniStop surgery. **Methods:** This retrospective cohort included 174 women with stress urinary incontinence who underwent Altis (*n* = 118) or MiniStop (*n* = 56) surgery. Sling position along the urethral length was expressed as a percentage from the bladder neck (0%) to the external urethral meatus (100%), with 20–60% defined as the midurethral region. TPUS also assessed sling-to-urethra distance, coronal sling angles, and sling kinking. Clinical outcomes and complications were evaluated as secondary outcomes. **Results:** Slings were located within the midurethral region in 82.2% of Altis and 50.0% of MiniStop patients (*p* < 0.001). Coronal sling angles were smaller in the Altis group at rest and during straining (both *p* < 0.001). Sling-to-urethra distance and sling kinking did not differ significantly. Baseline leakage severity was greater in the Altis group, whereas concomitant pelvic reconstruction was more common in the MiniStop group. Questionnaire scores decreased within both groups. Recurrence occurred in 5.1% and 7.1%, respectively. **Conclusions:** Six-month TPUS identified distinct postoperative sling positions and coronal configurations in the two cohorts, with Altis slings more frequently located within the study-defined midurethral region. These findings characterize anatomical differences in the context of baseline and procedural differences and provide a basis for investigating their relationship with clinical outcomes.

## 1. Introduction

Stress urinary incontinence (SUI) is common in women and can substantially impair quality of life. Midurethral sling (MUS) surgery is an established treatment, with retropubic and transobturator approaches providing favorable long-term continence outcomes [1,2]. However, these procedures require trocar passage through the retropubic or obturator space and may be associated with bladder injury, groin or postoperative pain, voiding dysfunction, and mesh-related adverse events [2,3].

Single-incision mini-slings were developed to reduce route-related morbidity while maintaining midurethral support. They are inserted through a short vaginal incision without external skin incisions and avoid trocar passage through the retropubic or obturator space. Randomized evidence and systematic reviews suggest that contemporary single-incision mini-slings can achieve short- to medium-term continence outcomes comparable to those of standard MUS procedures, with potential advantages in operative time, postoperative pain, and recovery [4,5,6,7]. Nevertheless, direct comparisons between different third-generation mini-sling systems remain limited.

Altis and MiniStop are third-generation single-incision mini-slings used to treat female SUI. Clinical studies have reported favorable outcomes after Altis [8,9] and MiniStop [10,11], but direct comparisons of their postoperative sling position and dynamic sling configuration are lacking.

Transperineal ultrasound (TPUS) is a noninvasive imaging modality that can depict anatomical and dynamic changes after anti-incontinence surgery. It enables assessment of bladder-neck and urethral mobility [12,13], as well as sling position, the sling–urethra relationship, and dynamic sling behavior after surgery [14,15,16]. In our previous studies, TPUS parameters correlated with the Q-tip test for urethral hypermobility and were associated with de novo SUI after transvaginal mesh surgery [17,18]. These findings support the use of TPUS to characterize postoperative sonographic differences between mini-sling systems.

The present study therefore aimed primarily to compare sling position and dynamic sling configuration on 6-month TPUS after Altis and MiniStop procedures. Secondary objectives were to evaluate questionnaire scores, urodynamic parameters, perioperative complications, and recurrence. We hypothesized that the two systems would demonstrate different postoperative sonographic configurations.

## 2. Materials and Methods

### 2.1. Study Design and Population

This retrospective cohort study was conducted at Kaohsiung Medical University Chung-Ho Memorial Hospital. Women diagnosed with stress urinary incontinence (SUI) who underwent single-incision mini-sling surgery using either the Altis system (Coloplast Corp., Minneapolis, MN, USA) or the MiniStop system (APIS Technologies Sarl, Aubonne, Switzerland) between April 2020 and March 2023 were reviewed. The diagnosis of SUI was based on clinical symptoms, pelvic examination, and preoperative urodynamic evaluation in all patients. The choice of sling system was based on surgeon preference rather than randomized allocation.

Women were eligible if they had clinically significant SUI, underwent Altis or MiniStop sling placement, and had available preoperative and postoperative clinical data. Exclusion criteria were incomplete medical records, insufficient follow-up, active urinary tract infection at evaluation, neurogenic bladder, or another major neurological disorder affecting lower urinary tract function.

Postoperative follow-up was scheduled at 1, 3, 6, and 12 months after surgery and semiannually thereafter. Recurrence was defined as symptomatic SUI occurring during postoperative follow-up.

The study was approved by the Institutional Review Board of Kaohsiung Medical University Hospital (IRB No. KMUHIRB-E(I)-20260305), which waived the requirement for informed consent because of the retrospective design. The study was conducted in accordance with the Declaration of Helsinki.

### 2.2. Preoperative Urodynamic Assessment and Surgical Selection

All patients underwent preoperative urodynamic testing, and the findings were reviewed when determining suitability for sling surgery. Filling cystometry characterized bladder storage function. Detrusor contractility and possible bladder outlet obstruction were assessed using the voiding-phase pressure–flow findings, including the relationship between detrusor pressure and urinary flow. Patients in whom the overall preoperative assessment established detrusor underactivity or bladder outlet obstruction were considered unsuitable for sling surgery because of concern about postoperative urinary retention.

An elevated postvoid residual (PVR) volume was interpreted together with the clinical assessment and the other urodynamic findings. Possible difficulty voiding naturally in the examination setting, including anxiety or difficulty relaxing, was considered when interpreting the voiding episode. PVR alone was not used as the sole determinant of surgical suitability. These assessments guided eligibility for sling surgery itself rather than the selection of Altis versus MiniStop.

### 2.3. Surgical Procedures

All procedures were performed by experienced urogynecologic surgeons. Both systems were inserted through a short vaginal incision approximately 1 cm proximal to the external urethral meatus, with placement intended to provide midurethral support. Sling tension was adjusted intraoperatively based on surgical judgment to provide midurethral support while avoiding excessive urethral compression.

Concomitant pelvic procedures, including cervical amputation, total vaginal hysterectomy, and transvaginal mesh surgery, were performed during the same operation when clinically indicated.

During the study period, a long-loop tape-releasing suture was routinely placed during sling implantation in both groups as part of institutional practice. The preplaced suture provided an option for early postoperative sling loosening when urinary retention was attributed to sling-related outlet obstruction and release was clinically indicated. Our group previously described this technique in patients receiving TVT-O, Solyx, or Ophira slings [19].

### 2.4. Transperineal Ultrasound Assessment

Three-dimensional transperineal ultrasound (TPUS) was performed 6 months postoperatively using a Voluson 730 Expert ultrasound system (GE Healthcare Ultrasound, Zipf, Austria) to assess sling position and dynamic sling configuration. Examinations were performed with the patient in the supine position using a transperineal approach. Examinations were performed by each patient’s operating surgeon, and the examiners were aware of the implanted sling type. Each patient underwent one examination session comprising assessment at rest and during the Valsalva maneuver. Each applicable parameter was measured once under the corresponding condition.

The assessed parameters were longitudinal sling position, sling-to-urethra distance, resting and straining sling angles, and sling kinking during straining. Longitudinal sling position was expressed as a percentage of the total urethral length, measured from the bladder neck (0%) to the external urethral meatus (100%). The midurethral region was defined as 20–60% of the urethral length, corresponding to the approximate anatomical region described by DeLancey [20]. This interval was used as an anatomical classification of sling position. Sling-to-urethra distance was defined as the shortest distance between the sling and urethra in the coronal plane. Resting and straining sling angles, defined as the angle formed by the two sling arms in the coronal plane, were measured at rest and during the Valsalva maneuver, respectively. Sling kinking during straining was defined as visible dynamic folding or angulation of the sling in the sagittal plane.

**Figure 1:**
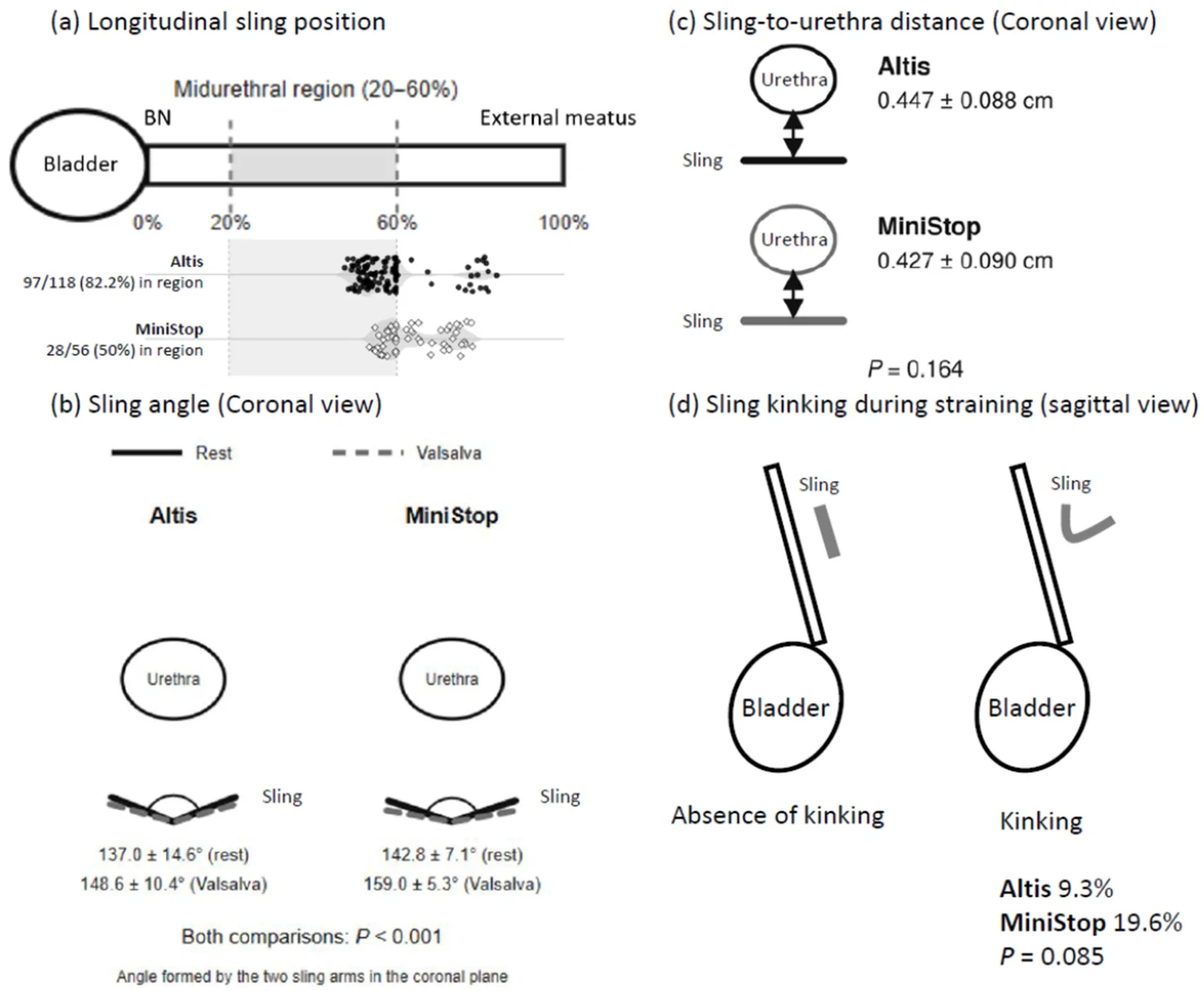
Schematic illustration of three-dimensional transperineal ultrasound measurements and observed sonographic findings 6 months after Altis or MiniStop single-incision mini-sling surgery. (**a**) Longitudinal sling position along the urethral length, measured from the bladder neck (0%) to the external urethral meatus (100%). Patient-level sling positions are shown beneath the schematic as black dots for Altis and open diamonds for MiniStop. The midurethral region was defined as 20–60% of the urethral length. The sling was located within this region in 97/118 (82.2%) patients after Altis and 28/56 (50.0%) patients after MiniStop surgery (*p* < 0.001). (**b**) Sling angle in the coronal plane, defined as the angle formed by the two sling arms. Coronal sling angles were measured at rest and during the Valsalva maneuver; group means are displayed. Both resting and straining angles were smaller after Altis than after MiniStop surgery (both *p* < 0.001). (**c**) Sling-to-urethra distance in the coronal plane, defined as the shortest distance between the sling and urethra (0.447 ± 0.088 cm after Altis vs. 0.427 ± 0.090 cm after MiniStop; *p* = 0.164). (**d**) Presence or absence of sling kinking in the sagittal plane during straining. Kinking was defined as visible folding or angulation of the sling and was observed in 9.3% of patients after Altis and 19.6% after MiniStop surgery (*p* = 0.085). Anatomical drawings are schematic and not to scale. Data are presented as mean ± standard deviation or *n*/*N* (%). BN, bladder neck; TPUS, transperineal ultrasound.

**Figure 2:**
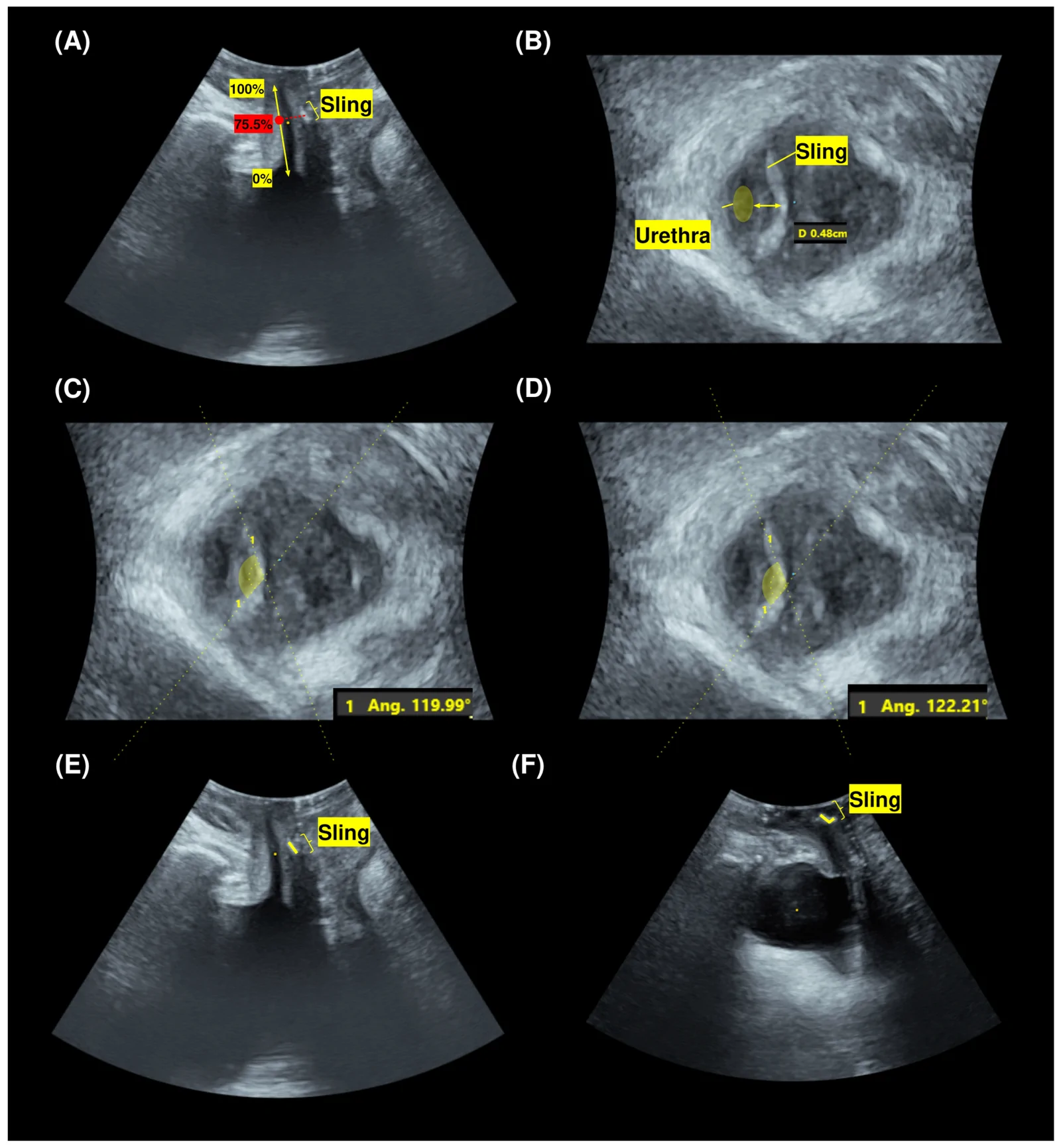
Representative postoperative transperineal ultrasound images illustrating the measured parameters. (**A**) Sling position along the urethral length, sagittal view. (**B**) Sling-to-urethra distance, coronal view. (**C**) Resting coronal sling angle. (**D**) Coronal sling angle during the Valsalva maneuver. (**E**) Absence of sling kinking during straining, sagittal view. (**F**) Sling kinking during straining, sagittal view. Displayed measurements illustrate individual examinations and are not group means; Yellow annotations indicate the urethral length and sling location in (**A**), and the urethra, sling, and sling-to-urethra distance in (**B**). In (**C**,**D**), the yellow dashed guide lines delineate the angle between the two sling arms, with the yellow shaded sectors highlighting the measured angle. In (**E**,**F**), yellow markers identify the sling.

The TPUS measurement methods and observed group findings are summarized schematically in Figure 1, and representative TPUS images are shown in Figure 2.

### 2.5. Clinical Assessment and Outcome Measures

Baseline demographic and clinical variables included age, parity, body mass index (BMI), 1-h pad-test weight, intrinsic sphincter deficiency (ISD), hormone therapy, menopausal status, hypertension, diabetes mellitus, previous hysterectomy, concomitant procedures, and follow-up duration.

Preoperative and 6-month postoperative assessments included pelvic examination, urodynamic testing, and interview-based assessment of urinary symptoms. Questionnaires were administered in Traditional Chinese. Overactive bladder symptoms were assessed using the Overactive Bladder Symptom Score (OABSS) [21]. Urinary symptom distress and incontinence-related impact were assessed using the Urogenital Distress Inventory-6 (UDI-6) and Incontinence Impact Questionnaire-7 (IIQ-7), respectively [22]. Urinary incontinence severity and its impact were assessed using the International Consultation on Incontinence Questionnaire–Urinary Incontinence Short Form (ICIQ-UI SF) [23]. Prolapse-related symptom distress was assessed using the Pelvic Organ Prolapse Distress Inventory-6 (POPDI-6), the prolapse subscale of the Pelvic Floor Distress Inventory-20 [24]. All 174 patients had complete preoperative and six-month postoperative questionnaire and urodynamic assessments (Altis, *n* = 118; MiniStop, *n* = 56).

Urodynamic parameters included maximum urinary flow rate (Qmax), postvoid residual volume, volume at first sensation (Vfst), maximum cystometric capacity (MCC), detrusor pressure at Qmax (PdetQmax), maximal urethral closure pressure (MUCP), functional urethral length, and urethral closure area (UCA).

Perioperative and postoperative complications were recorded, including bladder injury, rectal injury, blood transfusion, postoperative pain, urinary tract infection, voiding dysfunction, perineal hematoma, vaginal mesh extrusion, and bladder mesh extrusion. Postoperative pain was assessed using a visual analogue scale (VAS) on postoperative day 1.

### 2.6. Statistical Analysis

For between-group comparisons, continuous variables are presented as mean ± standard deviation (SD), and categorical variables as number (percentage). Continuous variables were compared using Student’s *t*-test or Welch’s *t*-test, and categorical variables using the chi-square test or Fisher’s exact test. For TPUS outcomes, continuous variables were compared using Welch’s *t*-test and categorical variables using Fisher’s exact test. All reported *p* values are two-sided, with *p* < 0.05 used as the threshold for statistical significance. To quantify the main TPUS comparisons, unadjusted effect estimates were calculated as Altis minus MiniStop. Mean differences and 95% confidence intervals (CIs) for continuous TPUS parameters were calculated from the reported means, SDs, and sample sizes using the Welch–Satterthwaite method. Percentage-point differences and 95% CIs for categorical TPUS outcomes were calculated using the Newcombe method based on Wilson score intervals without continuity correction. Intervals derived from rounded continuous summary statistics are approximate.

For questionnaire and urodynamic outcomes, within-group comparisons between the preoperative and six-month assessments were performed using the two-sided Wilcoxon signed-rank test. Descriptive mean changes (Δ) were calculated as the displayed six-month mean minus the displayed preoperative mean for the same participants. The Δ columns describe the direction and magnitude of the observed changes. Differences in within-group statistical significance were not interpreted as evidence of different treatment responses.

## 3. Results

The cohort comprised 174 women with SUI, including 118 in the Altis group and 56 in the MiniStop group. Age, parity, body mass index, menopausal status, hypertension, diabetes mellitus, and previous hysterectomy did not differ significantly between groups. Intrinsic sphincter deficiency was present in 22.0% of the Altis group and 10.7% of the MiniStop group (*p* = 0.094). Preoperative 1-h pad-test weight was higher in the Altis group (26.2 ± 34.8 vs. 5.72 ± 13.9 g; *p* < 0.001). Conversely, concomitant total vaginal hysterectomy and transvaginal mesh surgery were more common in the MiniStop group than in the Altis group (16.1% vs. 5.1%; *p* = 0.022 and 48.2% vs. 31.4%; *p* = 0.043, respectively). Follow-up ranged from 12 to 44 months after Altis and from 12 to 39 months after MiniStop surgery (Table 1).

At 6 months, a significantly greater proportion of patients in the Altis group had the sling located within the defined midurethral region than in the MiniStop group (97/118 [82.2%] vs. 28/56 [50.0%]; *p* < 0.001) (Figure 1a). The between-group difference was 32.2 percentage points (95% CI, 17.3–46.2). Mean sling angles were smaller in the Altis group both at rest (137.0 ± 14.6° vs. 142.8 ± 7.1°; *p* < 0.001) and during straining (148.6 ± 10.4° vs. 159.0 ± 5.3°; *p* < 0.001) (Figure 1b). The corresponding mean differences were −5.8° (95% CI, −9.0 to −2.6) at rest and −10.4° (95% CI, −12.8 to −8.0) during straining. Sling-to-urethra distance did not differ significantly between groups (0.447 ± 0.088 vs. 0.427 ± 0.090 cm; *p* = 0.164) (Figure 1c). Sling kinking during straining occurred in 11/118 (9.3%) Altis patients and 11/56 (19.6%) MiniStop patients, but the difference was not statistically significant (*p* = 0.085) (Figure 1d). These TPUS findings are summarized in Table 2 and Figure 1, and representative TPUS images are shown in Figure 2.

OABSS, UDI-6, IIQ-7, ICIQ-UI SF, and POPDI-6 scores decreased significantly from baseline to 6 months within each group (all *p* ≤ 0.03) (Table 3). Descriptive mean changes in ICIQ-UI SF were −9.5 in the Altis group and −5.7 in the MiniStop group; the corresponding OABSS changes were −2.0 and −2.5. Mean changes for all questionnaires are presented alongside the within-group *p* values in Table 3.

In the Altis group, Qmax decreased from 23.8 ± 10.9 to 20.3 ± 8.4 mL/s at six months (Δ, −3.5 mL/s; within-group *p* = 0.03). In the MiniStop group, PVR decreased from 121.7 ± 140.7 to 47.1 ± 45.8 mL (Δ, −74.6 mL; within-group *p* = 0.03). The other reported within-group *p* values were ≥0.05. Descriptive mean changes and the original within-group comparisons for all urodynamic parameters are presented in Table 4.

No bladder injury, rectal injury, or blood transfusion occurred in either group. No perineal hematoma, vaginal mesh extrusion, or bladder mesh extrusion was observed. Postoperative day-1 VAS scores did not differ significantly between the Altis and MiniStop groups (2.0 ± 1.4 vs. 1.8 ± 0.9; *p* = 0.51). Urinary tract infection occurred in 13/118 (11.0%) Altis patients and 7/56 (12.5%) MiniStop patients (*p* = 0.802). Voiding dysfunction occurred in no Altis patients and one MiniStop patient (1.8%; *p* = 0.322). During follow-up, recurrence occurred in 6/118 (5.1%) Altis patients and 4/56 (7.1%) MiniStop patients (*p* = 0.729) (Table 5).

## 4. Discussion

The principal sonographic finding of this study was a difference in longitudinal sling position on 6-month TPUS. The sling was located within the defined midurethral region (20–60% of the urethral length) in 82.2% of patients after Altis surgery and 50.0% after MiniStop surgery (*p* < 0.001), with the patient-level distribution shown in Figure 1a. The Altis group also had smaller coronal sling angles at rest and during straining. Sling-to-urethra distance did not differ significantly, while sling kinking during straining was numerically more frequent after MiniStop but did not reach statistical significance. Questionnaire scores improved significantly from baseline within each group, and recurrence and major perioperative or mesh-related complications were uncommon.

Randomized evidence and systematic reviews have reported comparable short- to medium-term continence outcomes for contemporary single-incision mini-slings and standard MUS procedures [4,5,6,7]. Major perioperative and mesh-related complications were uncommon in both groups, consistent with published reports, although this study was not designed to establish comparative safety.

Previous studies have reported favorable medium-term efficacy and safety after Altis placement [8], including in women with intrinsic sphincter deficiency [9]. Studies of MiniStop have likewise reported favorable clinical outcomes [10,11]. In the present study, questionnaire scores improved within both treatment groups and recurrence rates were low. However, baseline leakage severity differed substantially between groups. These within-group improvements and the descriptive mean changes do not establish equivalent or superior clinical efficacy. Moreover, changes in questionnaire scores, particularly POPDI-6, may have been influenced by the concomitant pelvic reconstructive procedures.

The incorporation of TPUS is a strength of this study. TPUS provides noninvasive visualization of sling location, the sling–urethra relationship, and dynamic behavior during straining [14,15,16]. Our previous work demonstrated associations between TPUS parameters and the Q-tip test for urethral hypermobility [17] and between postoperative TPUS findings and de novo stress urinary incontinence after transvaginal mesh surgery [18]. Lo et al. reported no significant change in MiniStop position or mobility between six and twelve months [25]. Their cohort excluded women with high residual urine or concomitant prolapse surgery, limiting direct comparison with the present population. Their dynamic urethral kinking measure also differs from the sling folding assessed here. O’Kane et al. used extended two-dimensional TPUS to distinguish retropubic from transobturator slings, with performance varying by observer experience [26]. Within this broader imaging context, the present study extends this imaging approach to a direct comparison of two third-generation single-incision mini-sling systems.

The patient-level distribution along the normalized urethral axis shown in Figure 1a illustrates the difference in postoperative sling position between groups. The 20–60% interval follows an anatomical description of the midurethra [20], rather than a validated threshold for clinical success. Selected sonographic measures of sling location have been associated with surgical success [16]. Yip et al. reported higher cure rates with middle-third TVT-O placement [27], whereas Solhaug et al. found no association between sling location and cure 10–20 years after MUS surgery [28]. These studies used different positional definitions and follow-up intervals; the geometric middle third is also distinct from our 20–60% anatomical interval. Total vaginal hysterectomy and transvaginal mesh surgery were more common in the MiniStop group and may have influenced postoperative pelvic-floor geometry. The clinical significance of the observed positional difference therefore remains to be determined.

Initial placement and subsequent positional change are possible contributors. Despite a common incision landmark, variation among surgeons in deployment trajectory, introducer handling, anchor seating, and tension adjustment, together with individual anatomy, may affect final sling position. Our previous comparison of TVT and TVT-O also documented differences in postoperative tape location [29]. Postoperative migration remains a possible explanation. The six-to-twelve-month findings reported by Lo et al. do not resolve whether positional changes occur earlier [25]. Serial TPUS beginning soon after implantation would help distinguish initial placement from subsequent change.

The smaller coronal sling angles in the Altis group indicate a more acute configuration of the two sling arms. Caramazza et al. reported an association between sling-arm angle and patient-reported improvement using transvaginal ultrasound [30], but this does not establish a clinically meaningful threshold for the present differences of 5.8° at rest and 10.4° during straining. Our previous assessment of urethral compression examined changes in urethral cross-sectional dimensions during straining [31], a different measure from sling-arm angle. The angular differences should therefore not be interpreted as evidence of greater urethral compression.

Sling kinking during straining occurred in 9.3% of Altis patients and 19.6% of MiniStop patients, but the difference was not statistically significant. This finding should be considered exploratory because event numbers were small. Visible folding or angulation of the sling should not itself be equated with urethral obstruction.

The urodynamic findings also require cautious interpretation. Qmax decreased significantly within the Altis group, whereas postvoid residual volume decreased within the MiniStop group. The reduction in postvoid residual volume in the MiniStop group may also have been influenced by the higher frequency of concomitant pelvic reconstructive procedures. Surgical suitability was determined from the overall preoperative clinical and urodynamic assessment, including voiding-phase pressure–flow findings, rather than PVR alone. Patients with established detrusor underactivity or bladder outlet obstruction did not proceed to sling surgery. Routine long-loop placement provided an option for early sling loosening if postoperative retention was attributed to sling-related obstruction. Our group previously described this technique in women receiving TVT-O, Solyx, or Ophira slings [19]. These isolated within-group findings do not demonstrate different physiological effects of the two sling systems.

This study has several limitations. Its retrospective, non-randomized design introduces potential selection bias, particularly because sling selection was based on surgeon preference. Baseline leakage severity and concomitant pelvic procedures differed between groups, and the analyses were not adjusted for these imbalances. The smaller MiniStop group and low event numbers limited precision for recurrence, complications, and sling kinking. Follow-up duration was not uniform, and TPUS was evaluated at a single postoperative time point. TPUS was performed by each patient’s operating surgeon, who was aware of sling type, with one measurement per condition; variation between examiners and unblinded assessment may influence the measurements. Intraobserver and interobserver reproducibility were not assessed. Prospective studies with serial imaging, covariate adjustment, and direct assessment of imaging–outcome relationships are needed.

## 5. Conclusions

TPUS performed six months after surgery identified a marked between-group difference in sling position along the urethral length, with a greater proportion of Altis slings located within the defined midurethral region. The groups also differed in coronal sling angles, whereas sling-to-urethra distance and kinking did not differ significantly. These findings characterize distinct postoperative sonographic configurations in the two clinical cohorts and provide a basis for investigating whether these anatomical differences are associated with clinical outcomes.

## Figures and Tables

**Table 1:** Clinical background in both groups. Values are given as mean ± standard deviation or *n* (%).

		Altis (*N* = 118)	MiniStop (*N* = 56)	*p*-Value
Age		57.7 ± 11.7	58.9 ± 11.5	0.54
Parity		2.4 ± 1.2	2.41 ± 1.1	0.74
BMI (kg/m^2^)		24.6 ± 2.9	24.39 ± 4.1	0.72
1-h Pad-test weight (g)		26.2 ± 34.8	5.72 ± 13.9	<0.001
Intrinsic sphincter deficiency		26(22.0)	6(10.7)	0.094
Past history	Hormone therapy	17(14.4)	15(26.8)	0.06
	Menopause	49(41.5)	24(42.9)	0.87
	Hypertension	15(12.7)	8(14.3)	0.81
	Diabetes Mellitus	25(21.2)	14(25)	0.57
	Hysterectomy	22(18.6)	7(12.5)	0.39
Concomitant surgery	Cervical amputation	5(4.2)	3(5.4)	0.71
	Vaginal Total Hysterectomy	6(5.1)	9(16.1)	0.022
	Transvaginal mesh (TVM)	37(31.4)	27(48.2)	0.043
Follow-up (months)		12–44	12–39	

Values are mean ± SD or *n* (%), except follow-up duration, which is the minimum–maximum range. BMI, body mass index; TVM, transvaginal mesh. *p* values refer to unadjusted between-group comparisons. Follow-up ranges are descriptive.

**Table 2:** Transperineal ultrasound assessment in both groups.

	Altis (*N* = 118)	MiniStop (*N* = 56)	*p*-Value	Difference (95% CI) ^†^
Sling located within the midurethral region *, *n* (%)	97 (82.2%)	28 (50.0%)	<0.001	+32.2 (17.3 to 46.2) pp
Sling-to-urethra distance (cm)	0.447 ± 0.088	0.427 ± 0.090	0.164	+0.020 (−0.009 to 0.049) cm
Resting angle (°)	137.0 ± 14.6	142.8 ± 7.1	<0.001	−5.8 (−9.0 to −2.6)°
Straining angle (°)	148.6 ± 10.4	159.0 ± 5.3	<0.001	−10.4 (−12.8 to −8.0)°
Sling kinking during straining, *n* (%)	11 (9.3%)	11 (19.6%)	0.085	−10.3 (−23.2 to 0.3) pp

Data are shown as *n* (%) or mean ± SD. Categorical variables were compared using Fisher’s exact test, and continuous variables were compared using Welch’s *t*-test. * Midurethral region = 20–60%. SD, standard deviation; CI, confidence interval; pp, percentage points; TPUS, transperineal ultrasound. The midurethral region is measured along the urethral length from the bladder neck (0%) to the external urethral meatus (100%). Assessments were performed six months after surgery. *p* values are unadjusted between-group comparisons. ^†^ Differences are Altis minus MiniStop. Categorical differences use percentage points and 95% Newcombe–Wilson CIs without continuity correction. Continuous differences use 95% Welch–Satterthwaite t CIs calculated from the reported means, SDs, and sample sizes; these intervals are approximate because the summaries are rounded. Original *p* values are retained.

**Table 3:** Changes in validated questionnaire scores before and after surgery in both groups. Values are given as mean ± standard deviation.

	Altis (*N* = 118)				MiniStop (*N* = 56)			
	Pre-op	Post-op	Mean Change *	*p*-Value	Pre-op	Post-op	Mean Change *	*p*-Value
OABSS	5.3 ± 3.6	3.3 ± 2.9	−2.0	<0.01	4.7 ± 3.2	2.2 ± 1.9	−2.5	0.03
UDI-6	38.4 ± 21.7	10.8 ± 10.6	−27.6	<0.01	32.1 ± 25.1	9.7 ± 11.3	−22.4	0.01
IIQ-7	39.3 ± 28.9	8.4 ± 13.9	−30.9	<0.01	31.5 ± 32.0	6.2 ± 11.0	−25.3	<0.01
ICIQ-UI SF	11.7 ± 5.7	2.2 ± 3.4	−9.5	<0.01	7.5 ± 4.5	1.8 ± 2.9	−5.7	<0.01
POPDI-6	6.3 ± 5.1	1.6 ± 2.5	−4.7	<0.01	8.00 ± 5.0	2.17 ± 2.6	−5.83	<0.01

* Mean change (Δ) is six-month minus preoperative and was calculated from the reported rounded means for the same participants at both time points. Mean changes are presented descriptively; the SDs shown apply to the individual assessment time points. OABSS, Overactive Bladder Symptom Score; UDI-6, Urogenital Distress Inventory-6; IIQ-7, Incontinence Impact Questionnaire-7; ICIQ-UI SF, International Consultation on Incontinence Questionnaire–Urinary Incontinence Short Form; POPDI-6, Pelvic Organ Prolapse Distress Inventory-6. Values at each assessment are mean ± SD. SD, standard deviation; Pre-op, preoperative; Post-op, six-month postoperative. *p* values refer to within-group paired comparisons between the preoperative and six-month assessments using the two-sided Wilcoxon signed-rank test. The Δ columns provide descriptive comparisons of the observed changes.

**Table 4:** Urodynamic parameters in both groups. Values are given as mean ± standard deviation.

	Altis (*N* = 118)				MiniStop (*N* = 56)			
	Pre-op	Post-op	Mean Change *	*p*-Value	Pre-op	Post-op	Mean Change *	*p*-Value
Qmax (mL/s)	23.8 ± 10.9	20.3 ± 8.4	−3.5	0.03	13.6 ± 7.9	17.1 ± 6.2	+3.5	0.18
PVR (mL)	54.5 ± 79.4	55.1 ± 71.0	+0.6	0.91	121.7 ± 140.7	47.1 ± 45.8	−74.6	0.03
Vfst (mL)	166.7 ± 90.5	161.0 ± 70.9	−5.7	0.67	185.4 ± 87.1	170.0 ± 70.2	−15.4	0.28
MCC (mL)	399.2 ± 121.0	385.0 ± 100.6	−14.2	0.59	382.3 ± 107.7	416.8 ± 105.7	+34.5	0.22
PdetQmax (cmH_2_O)	24.2 ± 20.2	21.9 ± 25.7	−2.3	0.69	22.8 ± 11.7	26.6 ± 25.2	+3.8	0.74
MUCP (cmH_2_O)	55.3 ± 21.3	49.4 ± 16.1	−5.9	0.05	65.7 ± 27.5	55.5 ± 26.7	−10.2	0.11
FUL (mm)	26.9 ± 6.6	25.7 ± 5.8	−1.2	0.49	27.2 ± 7.9	25.8 ± 6.9	−1.4	0.17
UCA (cmH_2_O·cm)	800.7 ± 382.0	760.0 ± 334.4	−40.7	0.90	930.0 ± 569.5	848.2 ± 494.1	−81.8	0.31

* Mean change (Δ) is six-month minus preoperative and was calculated from the reported rounded means for the same participants at both time points. Mean changes are presented descriptively; the SDs shown apply to the individual assessment time points. Qmax: maximum urinary flow rate; PVR: Postvoid residual volume; Vfst: volume at first sensation; MCC: maximum cystometric capacity; PdetQmax: detrusor pressure at Qmax; MUCP: maximal urethral closure pressure; FUL: Functional urethral length; UCA: urethral closure area. Values at each assessment are mean ± SD. SD, standard deviation; Pre-op, preoperative; Post-op, six-month postoperative. *p* values refer to within-group paired comparisons between the preoperative and six-month assessments using the two-sided Wilcoxon signed-rank test. The Δ columns provide descriptive comparisons of the observed changes.

**Table 5:** Perioperative and postoperative outcomes and complications in the two groups. Values are given as mean ± standard deviation or *n* (%).

	Altis (*N* = 118)	MiniStop (*N* = 56)	*p*-Value
**Intraoperative complications**			
Bladder injury	0	0	-
Rectal injury	0	0	-
Blood transfusion	0	0	-
**Postoperative complications**			-
Post-op day1 VAS scores	2.0 ± 1.4	1.8 ± 0.9	0.51 ^#^
Urinary tract infection	13 (11.0)	7 (12.5)	0.802 **
Voiding dysfunction	0	1 (1.8)	0.322 **
Perineal hematoma	0	0	-
Recurrence	6 (5.1)	4 (7.1)	0.729 **
**Mesh complications**			
Vaginal extrusion	0	0	-
Bladder extrusion	0	0	-

Post-op, Postoperative; VAS, visual analogue scale. ^#^ Student’s *t*-test; ** Fisher’s exact test.

## Data Availability

The data presented in this study are available from the corresponding author upon reasonable request. The data are not publicly available due to privacy and ethical restrictions.
